# Deciphering the Research Environment of Dengue Epidemiology: A Bibliometric Analysis

**DOI:** 10.7759/cureus.90531

**Published:** 2025-08-19

**Authors:** Logan Tempel, Eric G Yang, Vindhya N Reddy, Latha Ganti

**Affiliations:** 1 Biology, Florida Virtual School Flex, Orlando, USA; 2 Biology, Brown University, Providence, USA; 3 Medicine, Apollo Institute of Medical Sciences and Research, Hyderabad, IND; 4 Medical Science, Brown University, Providence, USA

**Keywords:** arbovirus research, bibliometric analysis, climate change, dengue epidemiology, neglected tropical diseases (ntds)

## Abstract

This bibliometric analysis presents a comprehensive evaluation of the contemporary medical literature concerning dengue epidemiology, with a particular emphasis on its global impact in the 21st century. Utilizing an informetric approach, the study offers a systematic overview of the research landscape, identifying trends and driving factors influencing scholarly output in this domain. A dataset comprising 2,982 peer-reviewed publications from the past decade was curated from the Web of Science Core Collection, using the keyword “Dengue Epidemiology” to ensure thematic relevance. Subsequent analysis was conducted using VOSviewer version 1.6.20 software to generate visual mappings based on variables including country of origin, keyword co-occurrence, affiliated academic institutions, and publication venues. The dominance of research output from non-endemic regions suggests a shift in global interest, potentially reflective of the expanding geographic distribution of *Aedes aegypti*, the primary vector of the dengue virus. This expansion may be influenced by climate change, facilitated by anthropogenic factors such as industrialization and increased global mobility. The concentration of literature within the disciplines of infectious diseases and public, environmental, and occupational health underscores the public health relevance of this arbovirus. These findings suggest that globalization, climate change, and evolving vector ecology are reshaping the geographic and academic focus of dengue research. If current environmental and developmental trajectories persist, dengue may become increasingly prevalent in previously unaffected regions, reinforcing its emergence as a growing global health threat in the 21st century.

## Introduction and background

Since the first virological evidence of the dengue virus in 1943 by Ren Kimora and Susumu Hotta, this arbovirus has remained at the forefront of recent epidemiological literature [1]. Not only does dengue have a prominent mortality rate of over 20% if left untreated, but the virus has also seen a substantial 10-fold increase in frequency between the start of the 21st century and 2019 [2]. While, if treated promptly, the mortality rate of the virus is relatively low-under one percent-an estimated 10,000 individuals around the world still die from the disease each year. Dengue is primarily transmitted through the bite of an infected *Aedes aegypti* mosquito, which primarily inhabits tropical and subtropical regions, although recent reports indicate that more northern areas have begun to see signs of dengue as well. This trend is supported by the record-breaking 14 million reported cases of the virus in 2024, over double the 6.5 million reported cases in 2023 [3]. 

Furthermore, the rapid increase in the case burden of the dengue virus poses considerable challenges to global populations, challenges that are only expected to accelerate in the following years. According to BMJ Global Health, if the prevalence of dengue fever continues to rise at its current rate of approximately 57% annually, yearly global infections will advance to an alarming 77 million yearly global infections from 2041 onwards [4]. This drastic growth is mainly attributed to the repercussions of climate change, urbanization, global connectedness, and overall surface warming. Dengue’s characteristics as an arbovirus and increasing temperatures from climate change in recent years enable *A. aegypti* to transmit the virus in increasingly northern latitudes that would have previously been uninhabitable. While dengue hotspots are most typically seen in traditionally tropical climates such as Southeast Asia, Latin America, and West Africa, particularly Burkina Faso, climate change-which has contributed to expanding the potential habitat of *A. aegypti* mosquitoes-has given rise to the virus throughout the United States (particularly in Florida, Texas, and Hawaii) and sporadic outbreaks in Central European nations, namely France, and the Mediterranean region [5]. The perpetual emergence of arboviruses, such as dengue fever, in increasingly northern latitudes, such as the United States and the Mediterranean, demonstrates worrisome conditions for public health and illustrates the plurality of the effects of climate change. The consequences of this are furthered by widespread immigration and international travel. Thus, the distribution of dengue is heavily entrenched within a variety of factors and serves as evidence of the detrimental impacts of globalization, with increased urban sprawl and unsustainable industrial processes directly contributing to recent outbreaks [6]. As human activity continues to fuel global warming through infrastructure and industrialization, the expansion of dengue, along with other arboviruses, becomes imminent. Dengue’s continued presence in traditional hotspots throughout tropical, equatorial regions and expansion into more northern countries spearheads the virus’s role as a prominent threat to public health for both current and future generations [7]. 

The objective was to characterize literature with regard to dengue epidemiology. This assessment is crucial to understanding the diverse research landscape of dengue epidemiology and its development in a warming climate.

## Review

Methods

Bibliometric analysis is a critical methodological approach for examining research trends and patterns within large datasets, offering robust quantitative models that elucidate the structure and evolution of academic literature [8]. Its multimodal framework enables detailed statistical assessments of influential authors, institutions, journals, and recurring keywords across publications [9]. 

Data were retrieved from the Web of Science Core Collection (WOS), chosen for its extensive and reputable index of peer-reviewed literature and for its compatibility with the VOSviewer version 1.6.20 software. The search query was "dengue epidemiology" (as a topic versus title word). The search was limited to the past 10 years. Only English language articles were included. Limiting the analysis to a single database minimized duplication bias and ensured a uniform citation architecture. The dataset encompassed publications from January 2016 to June 12, 2025, capturing nearly a decade of scholarly output, including original articles, reviews, and case reports relevant to dengue epidemiology. The final dataset comprised 2,982 publications, after any duplicates were removed. 

Citation data, including titles, authors, abstracts, keywords, publication years, journals, and DOI, were downloaded as tab-delimited files and uploaded into VOSviewer version 1.6.20 software. Next, thresholds were set. A minimum of five was used for minimum documents per country; a minimum of zero was set for citations per publication. The reason we set a minimum of zero for citations per publication is that all papers are included in the visualization, even if they have not been cited by others. It means that all publications in your dataset, regardless of whether they have received any citations or not, will be included in the analysis and visualization. It ensures that even very new or less widely cited publications are considered in the analysis. Newer papers might not have had time to accumulate many citations, but they could represent emerging areas of research. Setting the minimum to zero allows us to identify these early trends. A threshold of zero prevents us from excluding potentially important work in this area.

Finally, publications were analyzed for country of origin, annual publication frequency, keywords, affiliated institutions, author identity, journal of publication, and disciplinary classification. VOSviewer version 1.6.20 online software was utilized to generate visual bibliometric maps, enabling qualitative interpretation of spatial, temporal, and thematic trends in the dataset.

Results

**Figure 1:**
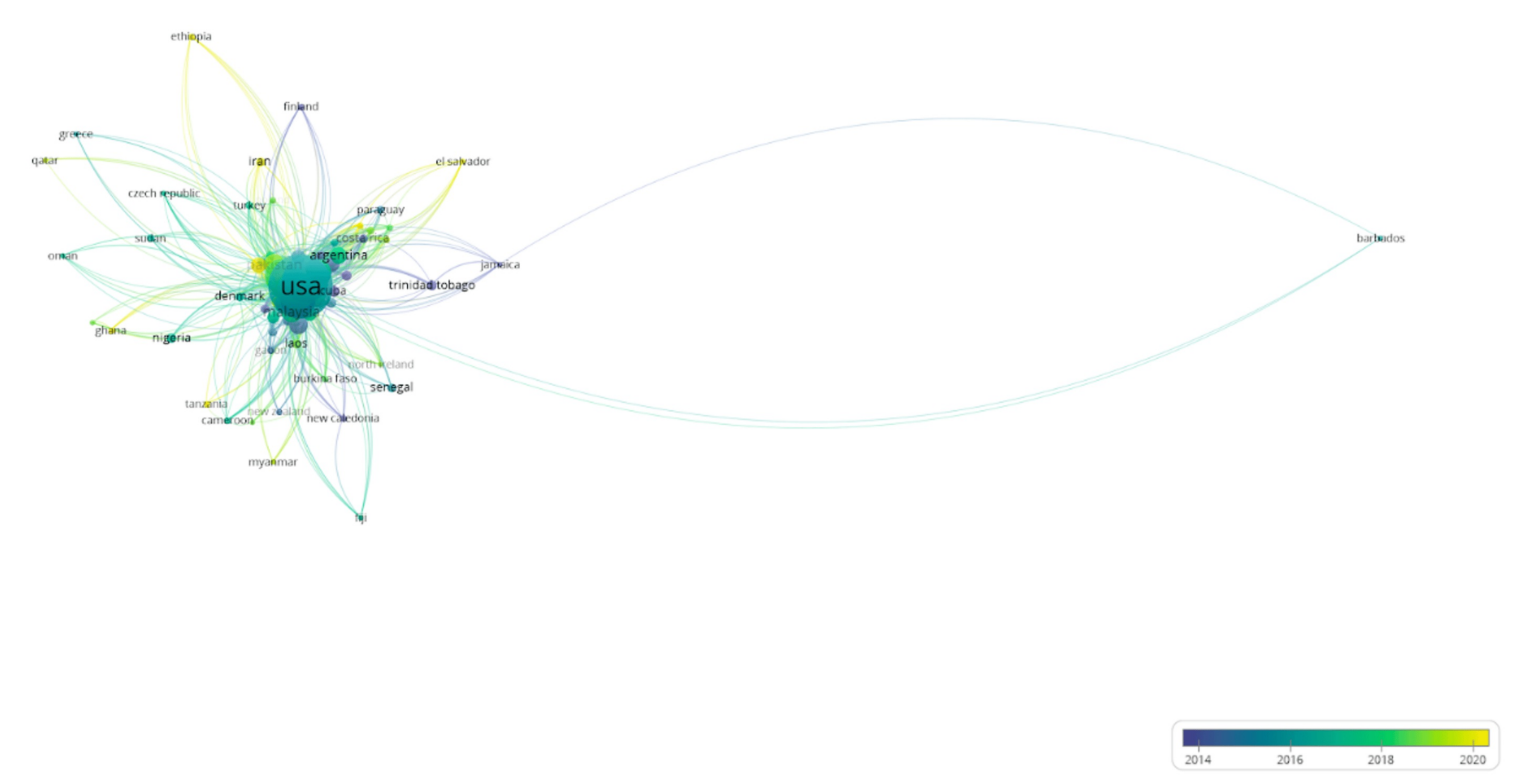
Dengue epidemiology publication data by country of origin The image was created by Logan Tempel using VOSviewer version 1.6.20 software.

Figure 1 is a visual representation of the dataset by exemplifying the frequency of publication data, organized by country of origin with respect to time. In this figure, the size of each circle denotes the frequency of publications on dengue epidemiology, while the color of each circle indicates when each work was published. Using the visualization, it is evident that the United States (USA) serves as the primary reference in scientific literature on dengue epidemiology from 2014 to 2020, with the majority of publications produced during 2017. Further, the figure illustrates distinct trends within the regions in which each work was published. While the United States easily attains the largest number of publications published, the data indicates that publications are still prevalent in tropical, equatorial regions-namely, Latin America (Argentina, Paraguay, Costa Rica, El Salvador), the Caribbean (Cuba, Trinidad and Tobago, Jamaica, Barbados), West Africa (Senegal, Cameroon, Ghana, Gabon, Burkina Faso), and Southeast Asia (Myanmar, Laos, Malaysia). 

The data illustrate a distinct presence of countries with diverse latitudes and climates, such as in Northern Europe (Northern Ireland, Denmark, and Finland) and the Mediterranean (Greece and Turkey). Further, publications are also clustered in the Pacific Islands (Fiji, New Caledonia, and New Zealand) and Central Asia (Iran and Pakistan); these regions also demonstrate the warmer temperatures of West Africa, the Caribbean, and South America while developing the diverse geographical origins of publications in the dataset. With respect to time, the data is not fully clustered, excluding scientific literature from Iran and Pakistan (2020), East Africa (Ethiopia, Tanzania-2020), and the Caribbean (Cuba, Jamaica, Trinidad and Tobago-2014).

**Figure 2:**
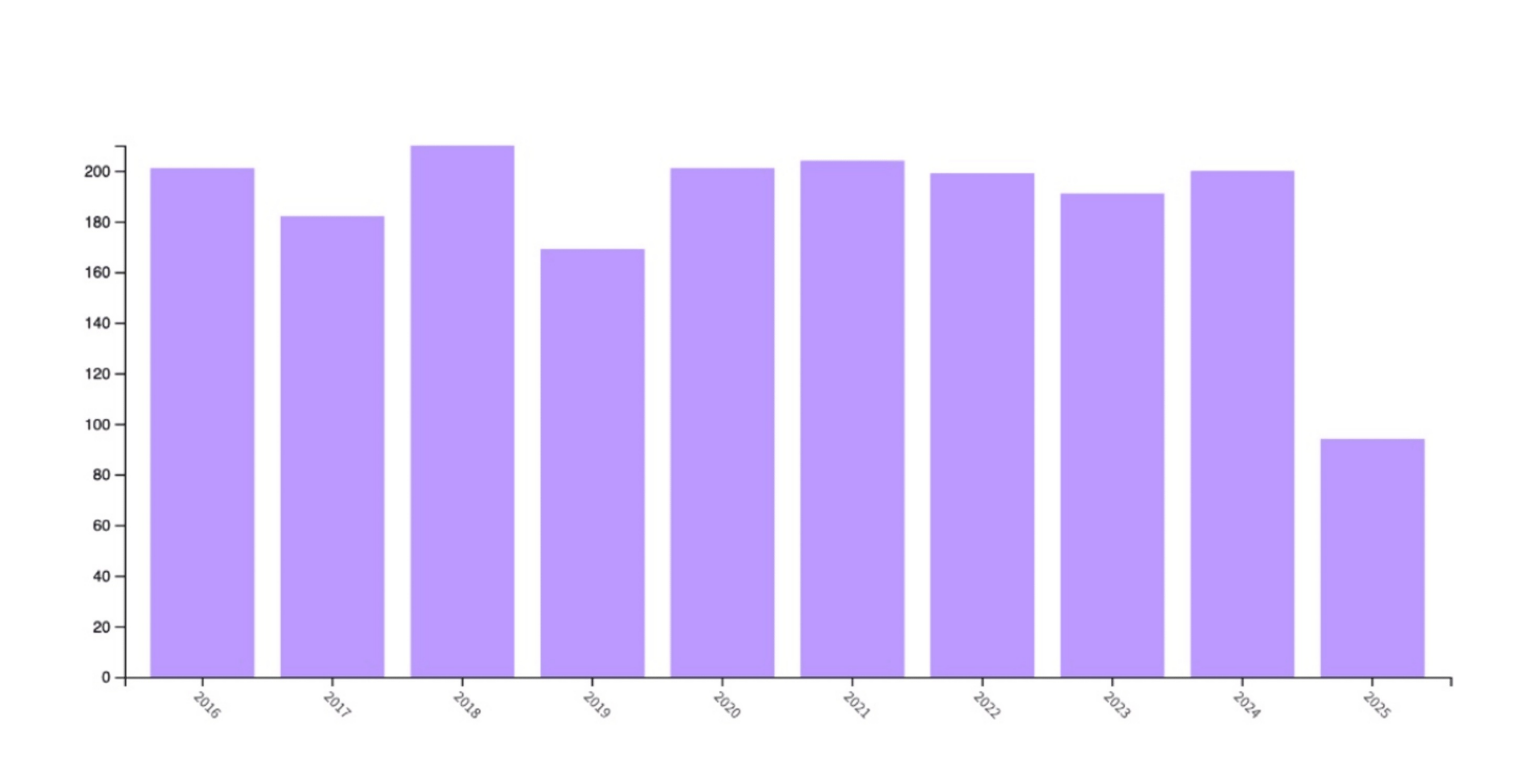
Dengue epidemiology publications per year (2016-2025) The image was created by Logan Tempel from Web of Science data.

Figure 2 conveys annual trends in scientific literature from 2016 to 2025 by the total number of publications each year. Figure 2 demonstrates that in the years between 2016 and 2025, the number of publications ranged from roughly 170 in 2019 to approximately 210 in 2018. Over the 10 years, there has been an observed average of 195 articles on dengue epidemiology per year. The data for 2025, with approximately 100 publications, is not fully complete, as the graph only compiled data until June 12, 2025. Assuming that the publication frequency in 2025 continues at a constant rate, there are expected to be roughly 200 articles by the year’s end, demonstrating continuity with the graph's stagnant trend, with minor yearly variation, by attaining a similar amount to the dataset’s average number of annual publications, 195.

**Figure 3:**
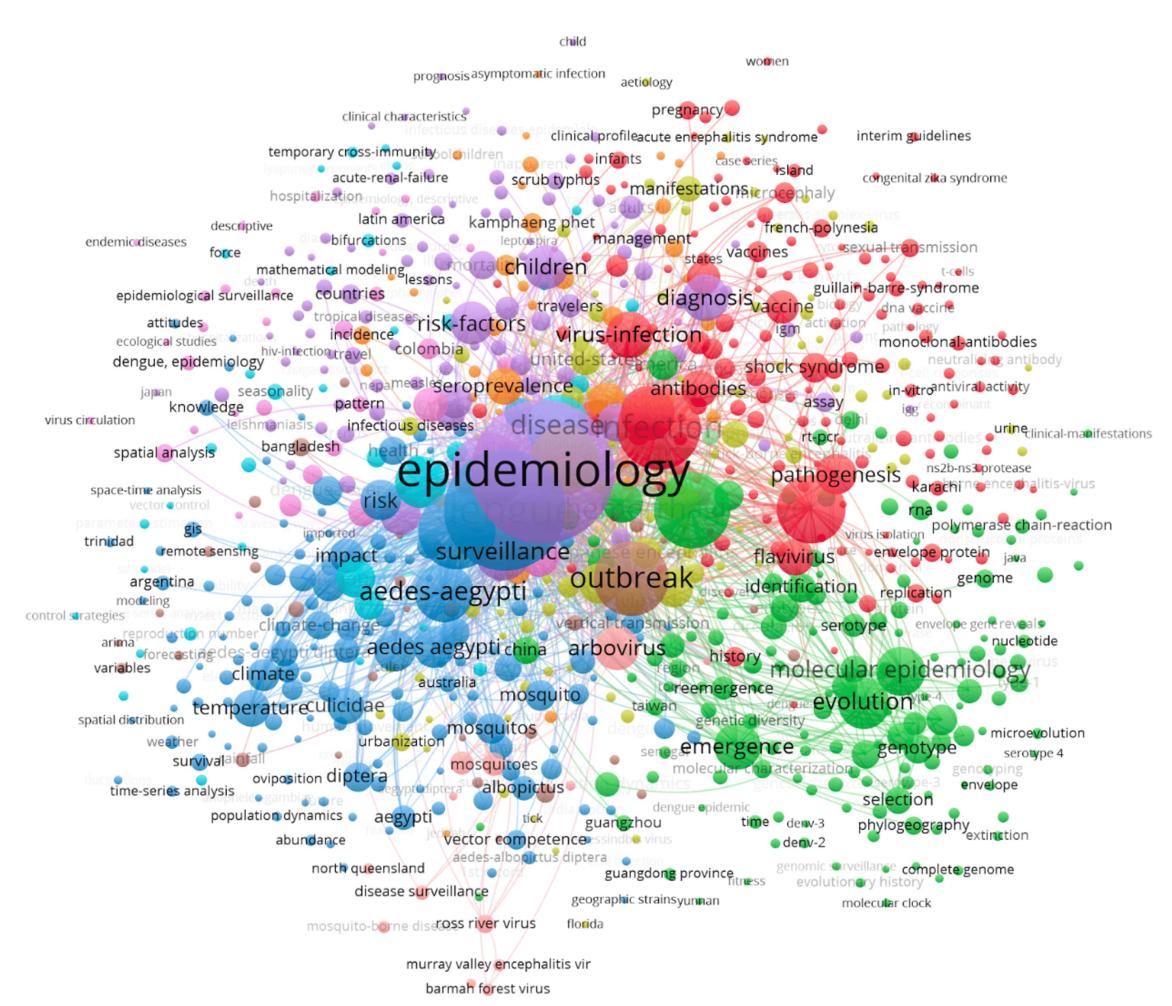
Frequency of keywords in dengue epidemiology literature The image was created by Logan Tempel using VOSviewer version 1.6.20 software.

Figure 3 visualizes the prevalence of certain keywords in scientific literature, where the size of a circle denotes a keyword's prevalence in the dataset. By analyzing the figure, it can be seen that the most common keywords found in publications are epidemiology, dengue, and hemorrhagic fever. Some additional relevant keywords found in the database include "disinfection," "outbreak," "surveillance," and "*A. aegypti*." Prominent keywords, e.g., "epidemiology," "dengue," and "arbovirus," occur frequently in the dataset due to how these keywords effectively describe how the majority of documents refer to dengue by its main characteristics, as well as how they reinforce that the epidemiology of the virus is a global concern. Additionally, specific keywords regarding locations, such as French Polynesia, Japan, China, Australia, Senegal, Taiwan, and Bangladesh, are prevalent due to their connection with traditional dengue hotspots. The data also indicates that keywords such as urbanization, climate change, and temperature are prominent when examining the causative factors in dengue epidemiology as they relate to factors linked to the virus, emphasizing its interconnected nature. The consistency of keywords such as "epidemiology," "outbreak," and "arbovirus" in 2,982 medical texts, along with those that relate to climate change and urbanization, helps to corroborate the theory that climate change is a propelling factor behind the evolution of dengue epidemiology.

**Figure 4:**
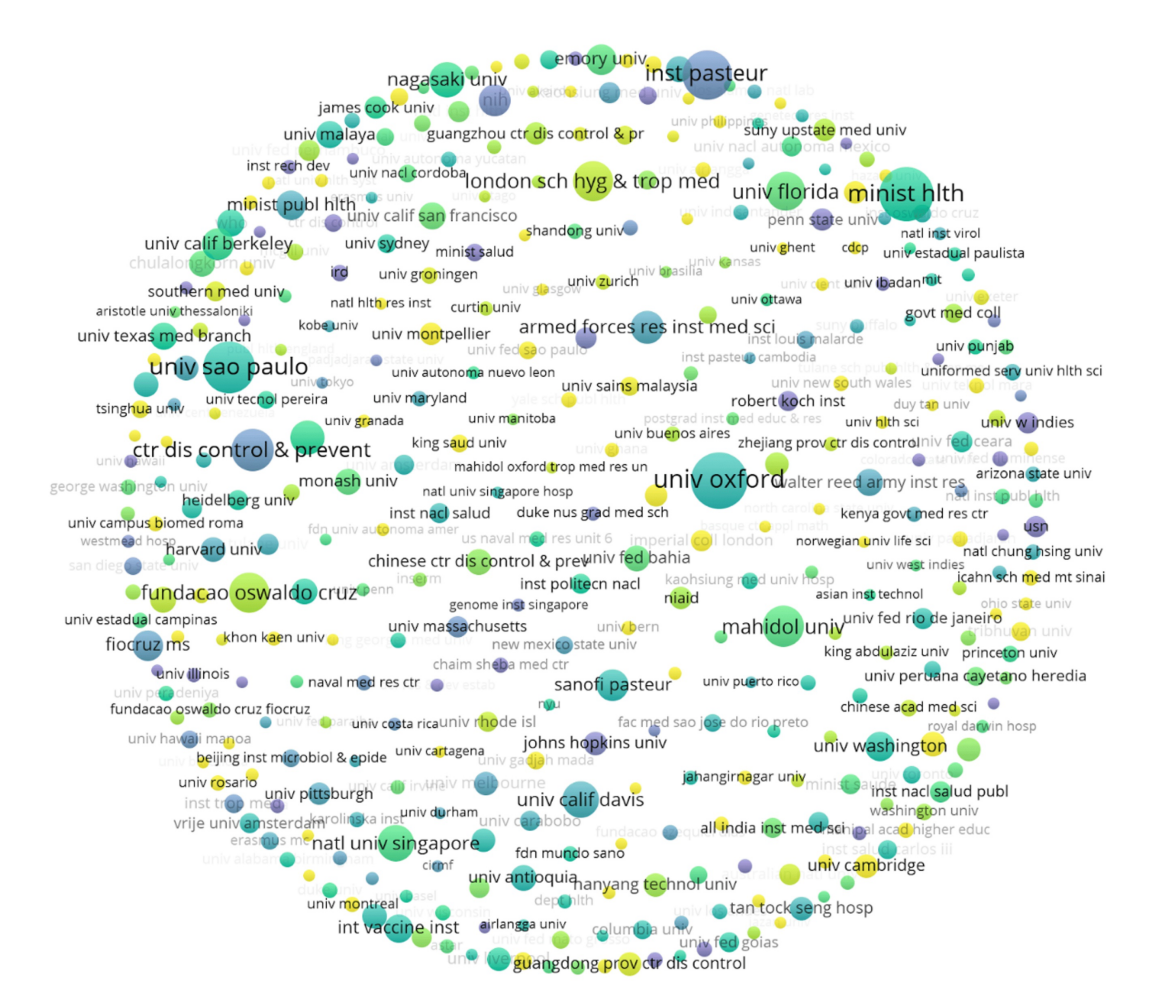
Frequency of research institutes in dengue epidemiology literature The image was created by Logan Tempel using VOSviewer version 1.6.20 software.

Figure 4 addresses the role that various notable institutions possess in publications on dengue epidemiology. As seen, the institutions that are most frequently published in the literature on dengue epidemiology are the University of Oxford, the University of São Paulo, the Ministry of Health, and the Institut Pasteur. Further, Figure 4 shows that these four institutions published the highest frequency of literature from 2016 to 2017; however, it is important to recognize that 2020 has the highest number of participating institutions, with data that is less clustered around a small group of institutions. It can also be seen that the United States and the United Kingdom possess the largest number of articles on dengue epidemiology in the dataset, by both the number of institutions and publications. Additionally, Brazilian, Chinese, and Western European institutions show significant contributions to the dataset.

**Figure 5:**
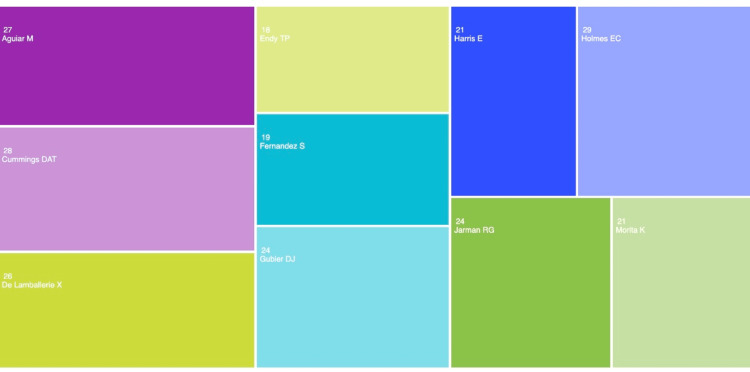
Frequency of dengue epidemiology publications by author The image was created by Logan Tempel from Web of Science data.

Figure 5 visualizes the bibliometric input of the top 10 most prominent authors in the dataset by the number of articles published. The figure reveals that the most prominent author in the dataset is E.C. Holmes with 29 publications (University of Sydney). The second and third most established authors in the dataset are DAT Cummings with 28 publications and M. Aguiar with 27 publications from the Johns Hopkins Bloomberg School of Public Health and the Basque Center for Applied Mathematics, respectively. The 10 most influential authors in this dataset have published an average of 23.7 publications on dengue epidemiology each. Further, it can be seen that the associated institutions of the top three most prominent authors in the dataset directly reflect the regional trends in Figure 1, where the United States, the United Kingdom, and Western Europe are the most recognized countries in the dataset. 

**Figure 6:**
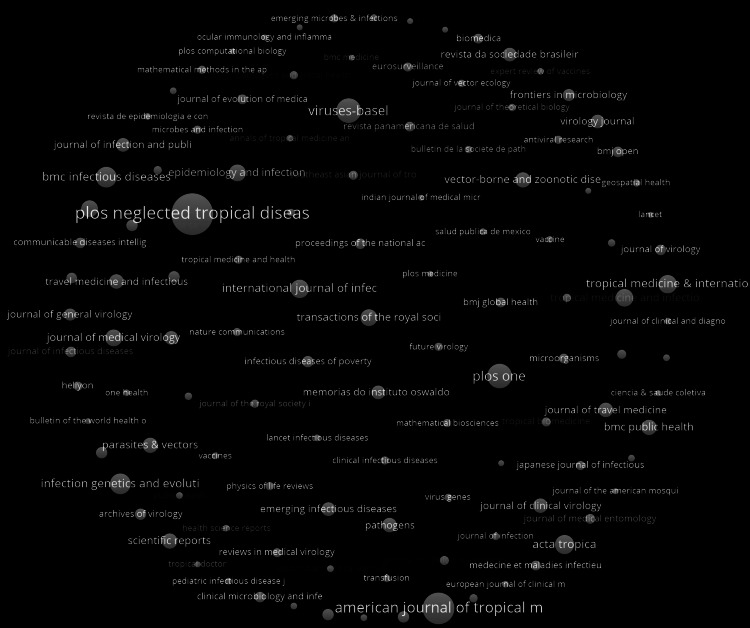
Frequency of medical journals in dengue literature The image was created by Logan Tempel using VOSviewer version 1.6.20 software.

Figure 6 addresses the impact of correlated medical journal publications within the research landscape of dengue epidemiology, where the size of a circle denotes the number of articles present in each respective medical journal. Some journals possess vastly more publications in the dataset than other participatory medical journals, with Public Library of Science (PLOS) Neglected Tropical Diseases being the most prominent in dengue epidemiological literature. Additionally, it can be seen that the American Journal of Tropical Medicine, Viruses-Basel, and PLOS One are the second, third, and fourth most prominent journals in the data, respectively. Similar to the results from Figure 5 and Figure 1, the United States continues to spearhead publication frequency on dengue epidemiology, with the role of Viruses-Basel in the dataset also reflecting how European medical journals are prevalent in the dataset, in addition to the U.S.

Discussion

The findings from this bibliometric study highlight the shifting landscape of dengue epidemiology research over the past decade. While the United States remains the predominant contributor, likely due to its robust academic infrastructure and access to research funding, significant input is also observed from tropical regions where dengue remains highly endemic. 

Although the prevalence of dengue fever has recently been seen in the United States in Florida, Texas, Hawaii, and Arizona, which may contribute to increased academic research and funding efforts, other tropical areas remain vastly more affected by the arbovirus [10]. This is seen throughout Latin America, West Africa, and Southeast Asia, as countries such as Brazil represent over 6 million confirmed dengue cases midway through 2024, growing to 9.6 million by the year's end and accounting for 68% of globally reported cases [11,12].

Despite their lower disease burden, non-endemic nations, including the United Kingdom, France, and China, are emerging as major centers of dengue research. This may reflect increasing concern over the geographic spread of *Aedes aegypti*-a phenomenon possibly accelerated by climate change and international travel. Indeed, the United States experienced dengue outbreaks in over 30 states in 2025, while Europe reported multiple outbreaks in France, Italy, and Spain in 2023.

This trend aligns with historical data showing that from 1872 to 2015, countries outside traditional endemic zones (e.g., the U.S., U.K., France, and Japan) produced 38% of dengue-related literature, while tropical countries (e.g., Brazil, Thailand, India, and Malaysia) contributed 25% [13]. The current study actually focuses on the period where these data end, namely 2016-2025. The predominance of institutions and authors from non-endemic areas likely reflects both scientific capacity and a growing recognition of dengue’s potential to emerge in temperate zones.

The trend of authors, institutions, and research that are not associated with traditional regions of dengue outbreaks is reinforced by how Derek A.T. Cummings, an American, and Maíra Aguiar, a Spaniard, were the most influential authors in the analysis despite being based in non-endemic regions. These authors have a relatively high number of publications on dengue epidemiology, with a total of 56 among them. This trend is further supported by how the most impactful medical journals in the dataset, namely PLOS Neglected Tropical Diseases and Viruses-Basel, are also sourced in northern latitudes (the United States and Switzerland), regions atypical of dengue outbreaks. Coupling this finding with the recent dengue outbreaks in the U.S., which spanned 32 of the 51 states in 2025, and the similar presence in Europe, with over a dozen outbreaks in 2023 in France, Italy, and Spain, it is clear that areas non-traditionally associated with dengue are becoming increasingly vulnerable to the arbovirus [14]. 

While areas outside the typical tropical climates of dengue often have more resources and infrastructure, the record-breaking 14.1 million reported dengue cases in 2024, along with recent outbreaks in the United States and Western Europe, signal that there are factors actively expanding the distribution of the arbovirus [15]. The frequency of dengue in 2024 is possibly linked to the repercussions of global warming, as increasing surface temperatures-particularly in recent decades with fossil fuel emissions-can create new habitable territories for *A. aegypti* [16]. 

Environmental factors such as global warming, urbanization, and industrial emissions may be central to this shift. Surface temperature increases of 1.59°C since pre-industrial times have created more favorable environments for *A. aegypti* in regions previously inhospitable to the mosquito vector [17]. Projections suggest climate change could increase global dengue incidence by 18%-and up to 27% in some regions [18].

Furthermore, global mobility plays a critical role in viral transmission. Over 16.3 million U.S. tourists visit the Caribbean annually, while Western Europe receives the highest immigration inflows globally. Although travel does not inherently increase infection risk, it facilitates viral dispersion from endemic to non-endemic areas [19,20]. These factors may partially explain the high volume of dengue-related publications from high-income countries.

Limitations

This study is not without limitations. First, the analysis relied exclusively on the Web of Science Core Collection, introducing potential selection bias by excluding other prominent databases such as PubMed, Scopus, and JSTOR. Moreover, the dataset only included English-language publications, potentially overlooking significant research published in native languages of highly affected regions, such as Brazil, Thailand, or Vietnam. This introduces language bias and may skew the global research representation.

Additionally, the bibliometric approach captures quantity, not quality, of publications. Citation metrics or impact factors were not assessed in this analysis. Lastly, the limited date range (2016-2025) may not capture long-term trends in dengue epidemiology, nor historical shifts preceding the current period of climatic and geopolitical change.

Future investigations should incorporate multilingual, multi-database searches and explore thematic subdomains of dengue epidemiology, such as serotype evolution, vector control strategies, and vaccine development. Regional analyses with historical context will be essential in fully understanding the complex dynamics influencing dengue’s spread and the scholarly response to it.

## Conclusions

Dengue fever has continually posed a severe threat to public health since its inception in the French West Indies in 1635. Historically, the virus has been present in warm, tropical, and subtropical regions where the climate is an ideal habitat for *A. aegypti*. However, in recent decades, a distinct trend has begun to emerge: the presence and awareness of dengue fever have diffused into northern latitudes not traditionally susceptible to the arbovirus. The emergence and continuous role of the arbovirus in the United States, Western Europe, China, and Japan have led to these developed, modernized nations spearheading the development of dengue epidemiology’s research landscape, showing a distinct evolution in the virus’s research distribution. While the disproportionately high amount of publications from the United States and Western Europe may be due to discrepancies in research institutions and funding compared to developing nations in tropical climates, climate change and international tourism are also influential factors. As the global surface temperature increases, the *A. aegypti* mosquito expands its survivable range, exposing more individuals to dengue. 
